# Supplementary material for: The Density of Group I mGlu5 Receptors Is Reduced along the Neuronal Surface of Hippocampal Cells in a Mouse Model of Alzheimer’s Disease
Source: Int J Mol Sci. 2021 May 30;22(11):5867. doi: 10.3390/ijms22115867 (PMC8199018; doi:10.3390/ijms22115867)
Supplement: Supplementary file 1 [file ijms-22-05867-s001.zip › ijms-1227956-s001.pdf]

SUPPLEMENTARY MATERIAL

The density of Group I mGlu$_5$ receptors is reduced along the neuronal surface of hippocampal cells in a mouse model of Alzheimer´s disease

Martín-Belmonte et al.

Supplementary Material & Methods

Immunohistochemistry for light microscopy

Immunohistochemical reactions at the light microscopic level were carried out using the immunoperoxidase method as described earlier [1,2]. Briefly, sections were incubated in 10% normal goat serum (NGS) diluted in 50 mM Tris buffer (pH 7.4) containing 0.9% NaCl (TBS), with 0.2% Triton X-100, for 1 h. Sections were incubated in anti-mGlu$_5$ (1–2 µg/ml diluted in TBS containing 1% NGS), followed by incubation in biotinylated goat anti-rabbit IgG (Vector Laboratories, Burlingame, CA) diluted 1:200 in TBS containing 1% NGS. Sections were then transferred into avidin–biotin–peroxidase complex (ABC kit, Vector Laboratories). Bound peroxidase enzyme activity was revealed using 3,3´-diaminobenzidine tetrahydrochloride (DAB; 0.05% in TB, pH 7.4) as the chromogen and 0.01% H_2O_2 as the substrate. Finally, sections were air-dried and mounted prior to observation in a Leica photomicroscope (DM2000) equipped with differential interference contrast optics and a digital imaging camera.

Supplementary Figures

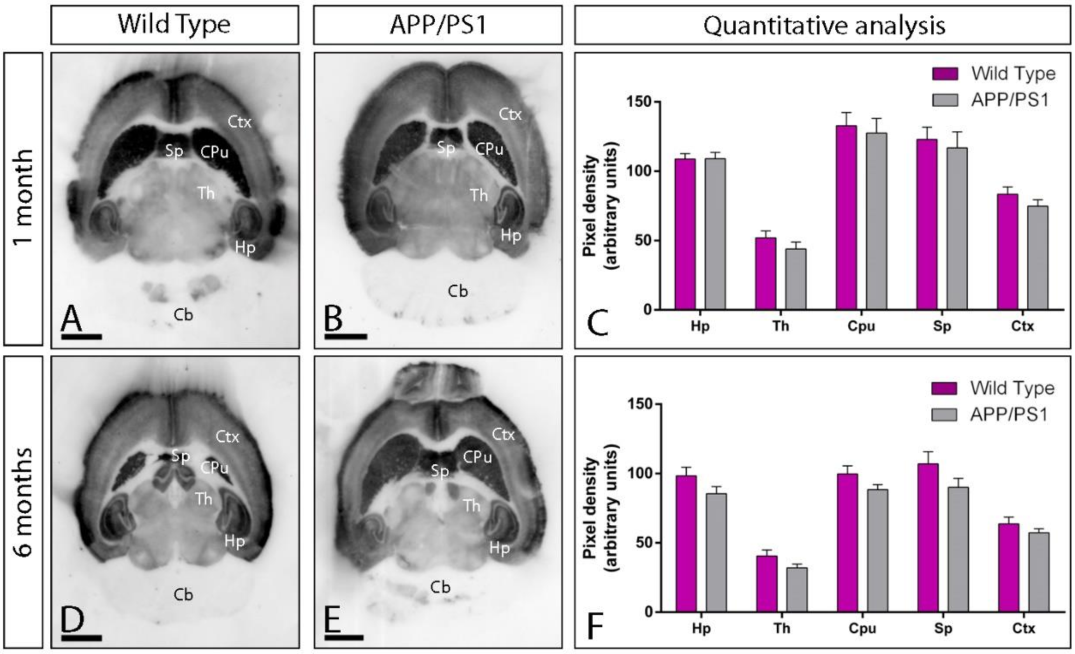

Supplementary Figure 1. ***Regional expression of mGlu$_5$ in the brain in wild type and APP/PS1 mice at early stages***. (A-I) The mGlu$_5$ expression was visualised in histoblots of horizontal brain sections at 1 and 6 months of age in wild type and APP/PS1 mice using an affinity-purified anti-mGlu$_5$ antibody. The expression of mGlu$_5$ in different brain regions was determined by densitometric analysis of the scanned histoblots. The expression of mGlu$_5$ revealed marked region-specific differences, with strongest immunoreactivity in the hippocampus (Hp), caudate putamen (CPu) and septum (Sp) and moderate labelling in the cortex (Ctx) and thalamus (Th). The weakest expression level was detected in the cerebellum (Cb). Densitometric analysis showed no differences in mGlu$_5$ expression in APP/PS1 mice compared to age-matched wild type controls at the two ages employed. Error bars indicate SEM. Scale bars: 0.2 cm.

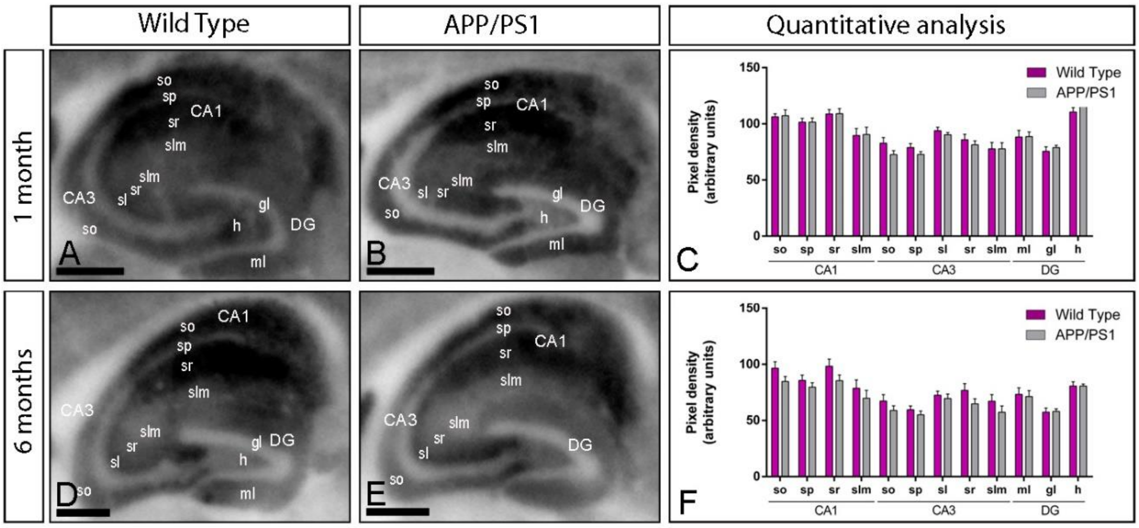

Supplementary Figure 2. ***Hippocampal expression and distribution of mGlu$_5$ in wild type and APP/PS1 mice at early stages***. (A-I) The mGlu$_5$ expression was visualised in histoblots of horizontal brain sections at 1 and 6 months of age in wild type and APP/PS1 mice using an affinity-purified anti-mGlu$_5$ antibody. The expression of mGlu$_5$ in different brain regions was determined by densitometric analysis of the scanned histoblots. Expression for mGlu$_5$ was strong in all dendritic layers of the CA1 and CA3 region and DG, with the *strata oriens* (so) and *radiatum* (sr) of the CA1 region showing the highest expression levels. A more moderate expression was observed in the *stratum lacunosum-moleculare* (slm) of CA1, the *strata oriens* (so), *radiatum* (sr) and *lacunosum-moleculare* (slm) of CA1, and the molecular layer (ml) and hilus (h) of the DG. showing the lowest expression level. Densitometric analysis showed no differences in mGlu$_5$ expression in APP/PS1 mice compared to age-matched wild type controls at the two ages employed. Error bars indicate SEM. Scale bars: 0.05 cm.

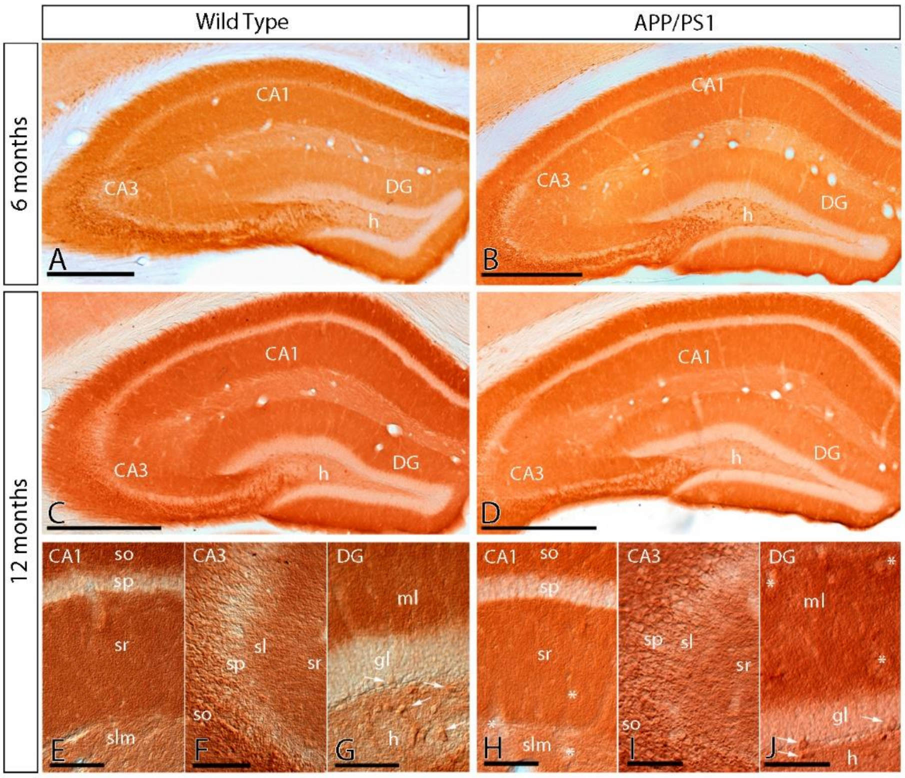

Supplementary Figure 3. ***Regional and cellular distribution of mGlu$_5$ in wild type and APP/PS1 mice***. (A-J) Immunoreactivity for mGlu$_5$ in the hippocampus of wild type and APP/PS1 mice at 6 and 12 months of age using a pre-embedding immunoperoxidase method at the light microscopic level. In the CA1 and CA3 regions and dentate gyrus (DG), intensity of mGlu$_5$ immunoreactivity was very similar both in the wild type and the APP/PS1 mice, regardless of accumulation of amyloid plaques (asterisks). Immunoreactivity for mGlu$_5$ was strongest in the *strata oriens* (so) and *radiatum* (sr) of the CA1 region. The *stratum lacunosum-moleculare* (slm) of the CA1 area, *strata oriens*, *radiatum* and *lacunosum-moleculare* of the CA3 region and molecular layer (ml) of the dentate gyrus were labelled less intensely. The *stratum lucidum* (sl) of CA3 and the hilus (h) of the dentate gyrus were only weakly labelled. Immunoreactivity for mGlu$_5$ was also detected in interneurons in the granule cell layer and mossy cells in the hilus (white arrows). No differences in mGlu$_5$ regional and cellular immunoreactivity was observed in APP/PS1 mice compared to age-matched wild type controls at the two ages employed. *Abbreviations*: sp, *stratum pyramidale*; gl, granule cell layer. Scale bars: A-D, 200 µm; E-J, 100 µm.

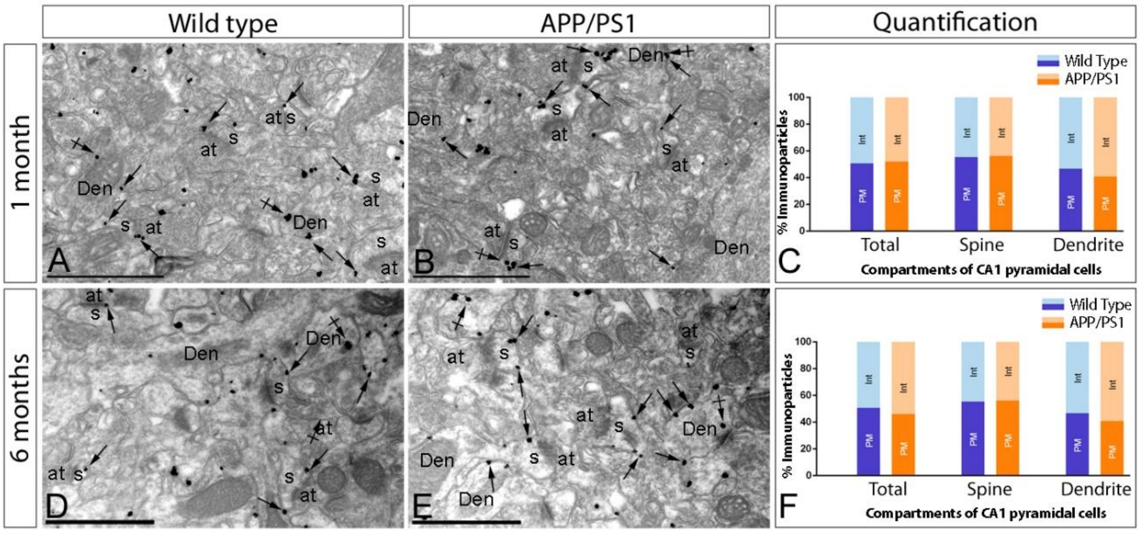

Supplementary Figure 4. ***Intracellular distribution of mGlu$_5$ in the CA1 region of APP/PS1 mice at early stages***. (A-F) Electron micrographs showing immunoparticles for mGlu$_5$ in the *stratum radiatum* of the CA1 region at 1 and 6 months of age in wild type and APP/PS1 mice, as detected using a pre-embedding immunogold technique. At the two ages, both in wild type and APP/PS1 mice, mGlu$_5$ immunoparticles were mostly located at the extrasynaptic membrane (arrows) of dendritic shafts (Den) and dendritic spines (s) of pyramidal cells, and less frequently at intracellular sites (arrowheads). (C, F) Quantitative analyses showed no changes between wild type and APP/PS1 mice in the frequency of mGlu$_5$ the plasma membrane and cytoplasmic sites. Scale bars: A,B,D,E: 1 µm.

References

1. Luján, R.; Nusser, Z.; Roberts, J.D.B.; Shigemoto, R.; Somogyi, P. Perisynaptic Location of Metabotropic Glutamate Receptors mGluR1 and mGluR5 on Dendrites and Dendritic Spines in the Rat Hippocampus. *Eur. J. Neurosci.* **1996**, *8*, 1488–1500, doi:10.1111/j.1460-9568.1996.tb01611.x.

2. Luján, R.; Roberts, J.D.B.; Shigemoto, R.; Ohishi, H.; Somogyi, P. Differential plasma membrane distribution of metabotropic glutamate receptors mGluR1α, mGluR2 and mGluR5, relative to neurotransmitter release sites. *J. Chem. Neuroanat.* **1997**, *13*, 219–241, doi:10.1016/S0891-0618(97)00051-3.
